# Identification of DNA-Dependent Protein Kinase Catalytic Subunit (DNA-PKcs) as a Novel Target of Bisphenol A

**DOI:** 10.1371/journal.pone.0050481

**Published:** 2012-12-05

**Authors:** Yuki Ito, Takumi Ito, Satoki Karasawa, Teruya Enomoto, Akihiro Nashimoto, Yasuyoshi Hase, Satoshi Sakamoto, Tsuneyo Mimori, Yoshihisa Matsumoto, Yuki Yamaguchi, Hiroshi Handa

**Affiliations:** 1 Graduate School of Bioscience and Biotechnology, Tokyo Institute of Technology, Midori-ku, Yokohama, Japan; 2 Solutions Research Laboratory, Tokyo Institute of Technology, Midori-ku, Yokohama, Japan; 3 Department of Rheumatology and Clinical Immunology, Graduate School of Medicine, Kyoto University, Sakyo-ku, Kyoto, Japan; 4 Research Laboratory for Nuclear Reactors, Tokyo Institute of Technology, Meguro-ku, Tokyo, Japan; University of Massachusetts Medical School, United States of America

## Abstract

Bisphenol A (BPA) forms the backbone of plastics and epoxy resins used to produce packaging for various foods and beverages. BPA is also an estrogenic disruptor, interacting with human estrogen receptors (ER) and other related nuclear receptors. Nevertheless, the effects of BPA on human health remain unclear. The present study identified DNA-dependent protein kinase catalytic subunit (DNA-PKcs) as a novel BPA-binding protein. DNA-PKcs, in association with the Ku heterodimer (Ku70/80), is a critical enzyme involved in the repair of DNA double-strand breaks. Low levels of DNA-PK activity are previously reported to be associated with an increased risk of certain types of cancer. Although the Kd for the interaction between BPA and a drug-binding mutant of DNA-PKcs was comparatively low (137 nM), high doses of BPA were required before cellular effects were observed (100–300 μM). The results of an *in vitro* kinase assay showed that BPA inhibited DNA-PK kinase activity in a concentration-dependent manner. In M059K cells, BPA inhibited the phosphorylation of DNA-PKcs at Ser2056 and H2AX at Ser139 in response to ionizing radiation (IR)-irradiation. BPA also disrupted DNA-PKcs binding to Ku70/80 and increased the radiosensitivity of M059K cells, but not M059J cells (which are DNA-PKcs-deficient). Taken together, these results provide new evidence of the effects of BPA on DNA repair in mammalian cells, which are mediated via inhibition of DNA-PK activity. This study may warrant the consideration of the possible carcinogenic effects of high doses of BPA, which are mediated through its action on DNA-PK.

## Introduction

**Figure 1:**
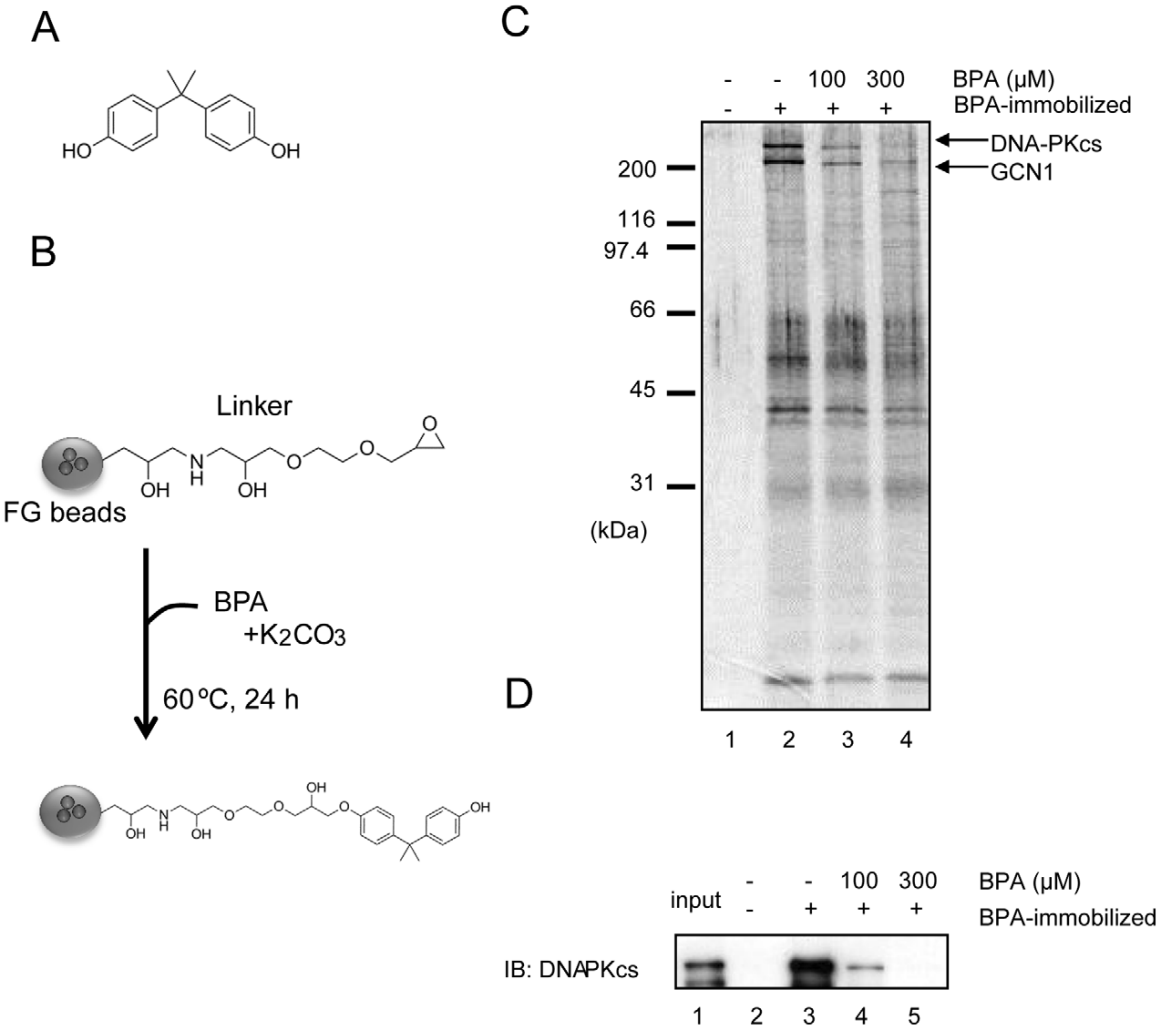
Identification of DNA-PKcs as a BPA-binding protein. (A) The structure of BPA. (B) Preparation of BPA-immobilized beads. (C) BPA-binding proteins were purified from MCF7 cell nuclear extracts using BPA-immobilized (+) or control (−) beads. Bound proteins were eluted with SDS sample buffer. In lanes 3 and 4, BPA was added to extracts before incubation with the beads. Eluted proteins were analyzed by silver staining (C) or immunoblotting (IB) (D).

Bisphenol A (BPA), 2,2-bis(4-hydroxyphenyl) propane (Fig. 1*A*), is widely used as a monomer in plastic products, including polycarbonate and other epoxy resins, which are used to coat food and drink cans, nursing bottles, dental sealants and many other items [1]. Annual worldwide production of BPA is approximately 3 million tons. However, when these plastics are treated with harsh detergents or acidic or high-temperature liquids, BPA can be released, resulting in daily (albeit unintentional) ingestion of BPA in foods and drinks, via inhalation, and/or by absorption through the skin [2].

BPA is an estrogenic chemical that interacts with human estrogen receptors (ER) [1]; however, binding of BPA to ER (and any subsequent hormonal activity) is extremely weak (1,000–10,000 times lower than natural hormones) [3]. BPA also shows reproductive and chronic toxicity [4]. The no observable adverse effect level for BPA was, however, reported *in vivo* even at intake of 50 mg/kg/day. Hence it was considered to be safe for packaging of food and beverages.

Recent studies illustrate the “low-dose effects” of BPA; for instance, an increase in the size and weight of the prostate in fetal mice [4]. The low-dose effects of BPA are thought to be mediated through steroid hormone receptors [5]; however, the weak interaction between BPA and ER does not support this hypothesis, which prompted us to examine whether BPA interacts with other receptors. Moreover, BPA interacts with a variety of cellular targets including the estrogen-related gamma receptor (ERRγ), a trans-membrane ER receptor called G-protein receptor 30, the aryl hydrocarbon receptor, the androgen receptor, the thyroid hormone receptor, the human glucocorticoid receptor [1], the non-classical membrane bound form of the ER [6], and protein di-sulfide isomerase [7].

Thus, BPA may have potentially negative effects on human health via its interaction with other nuclear receptors, although the underlying mechanisms remain unclear. Recently, the governments of several countries began to restrict the use of BPA. For instance, in 2010, the US Food and Drug Administration published in-depth studies on the risks of BPA and announced its support for a ban on the production of BPA-containing nursing bottles and feeding cups [8]. Canada became the first jurisdiction in the world to ban BPA and to declare it to be a toxic substance that may pose risks to human health [9]. However, in spite of the increased concern, BPA is still being used in many manufacturing processes.

Most studies to date focus on the low-dose effects of BPA, and there are no reports regarding its high-dose effects. However, nonfood exposure has been described; for instance, BPA can leech from polyvinyl chloride hoses into water and also from recycled or carbonless paper onto the skin [10]. This study shows that BPA may be present at higher levels than previously assumed. A study conducted on Chinese workers in BPA factories in 2009 revealed a 4-fold increase in the incidence of erectile dysfunction, reduced sexual desire, and overall dissatisfaction with their sex life in BPA-exposed workers compared with workers with no heightened BPA exposure [11]. This suggests that BPA may affect adult humans, although no other effects (apart from reduction in male sexual function) have been reported.

Here, we showed that DNA-dependent protein kinase catalytic subunit (DNA-PKcs) is a novel target for BPA. DNA-PKcs is a 470 kDa protein (4128 aa) and has phosphatidylinositol 3 (PI3)-kinase motifs near the carboxy terminus that constitutes the catalytic domain [12]. DNA-PKcs binds to the Ku heterodimer (Ku70/80) and forms DNA-PK complex, which is a serine/threonine kinase [13]. DNA-PK is activated by binding to the ends of double-stranded DNA, which is generated by ionizing radiation (IR) or by physiological processes such as V(D)J recombination, and is capable of phosphorylating a number of nuclear proteins including p53; although its physiological substrates still need to be clarified [14]. DNA-PKcs-deficient cells are more sensitive to ionizing radiation and DNA-damaging agents [15]–[17], which generate DNA double-strand breaks (DSBs), confirming the critical role played by DNA-PK in the recognition and repair of DSBs.

In the present study, we show that BPA binds directly to DNA-PKcs and inhibits its kinase activity at high concentrations both *in vitro* and in cultured cells. These findings provide a new insight into the “high-dose effects” of BPA, which are mediated through its action on DNA-PK.

## Results

### Binding of BPA to DNA-PKcs

**Figure 2:**
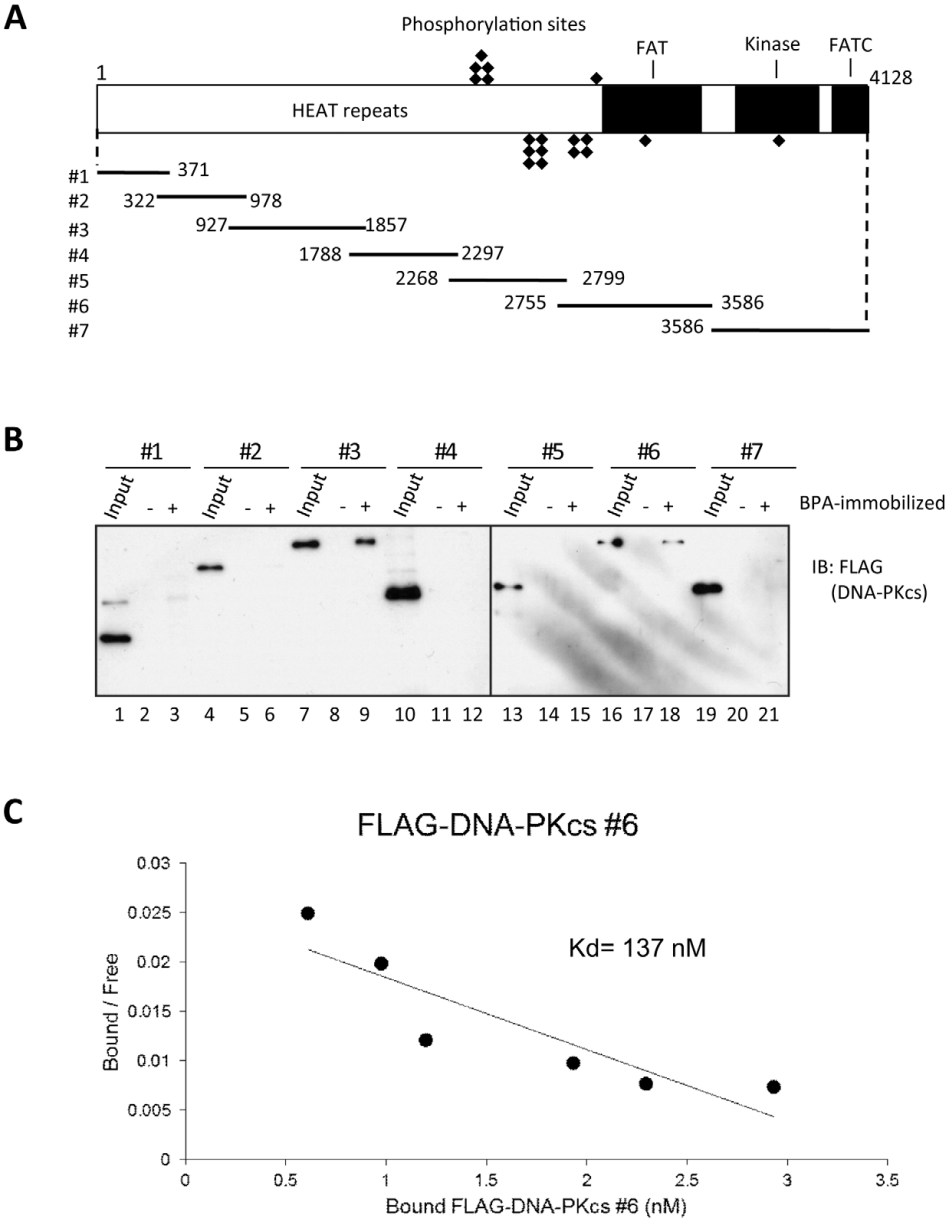
Binding of BPA to recombinant DNA-PKcs fragments *in vitro*. (A) Structure of DNA-PKcs and seven fragments. (B) Recombinant DNA-PKcs fragments (#1 to #7) were purified from 293T cells and incubated with BPA-immobilized (+) or control (−) beads. The input and bound proteins were immunoblotted with an anti-FLAG antibody. (C) The Kd for the binding of BPA to ΔDNA-PKcs #6 was calculated by Scatchard analysis.

To identify binding to BPA (Fig. 1*A*), affinity purification was performed using ferrite-glycidyl methacrylate (FG) beads (Fig. 1*B*). This method has several advantages over conventional affinity purification methods [18]. BPA was covalently conjugated to the beads, which were then incubated with nuclear extracts from MCF7 human breast cancer cells [19]. After washing five times, the beads were suspended in SDS sample buffer and the bound proteins eluted by boiling at 98°C for 5 min. The eluate fractions were subjected to SDS gel electrophoresis and silver staining. Two polypeptides with apparent molecular masses of >400 kDa and 300 kDa, respectively, were detected in the eluate from BPA-immobilized beads (Fig. 1*C* lane 2). When free BPA was added to the extracts before incubation with the beads, the yield of these proteins was substantially reduced (Fig. 1*C* lanes 3 and 4). These two polypeptide bands were subjected to proteolytic digestion and tandem mass spectrometry and were identified as DNA-dependent protein kinase catalytic subunit (DNA-PKcs; >400 kDa band) and general control of amino acid synthesis 1-like 1 (yeast) (GCN1; 300 kDa band). In the present study, we concentrated on DNA-PKcs as a potential binding protein for BPA. The identity of this protein was confirmed by immunoblotting using an antibody specific for DNA-PKcs (Fig. 1*D*). To determine whether the interaction between BPA and DNA-PKcs was direct, a recombinant, FLAG-tagged DNA-PKcs was constructed and then divided into seven fragments (ΔDNA-PKcs #1 to #7) (Fig. 2*A*). Each fragment was transiently expressed in 293T cells and purified using anti-FLAG M2 agarose beads. Each of the purified ΔDNA-PKcs fragments was then incubated with BPA-immobilized beads. Of the seven fragments, ΔDNA-PKcs#3 and ΔDNA-PKcs#6 showed strong binding to BPA (Fig. 2*B* lanes 7–9 and 16–18). These results suggest that the #3 and/or #6 regions are important for binding between BPA and DNA-PKcs. We measured the dissociation constant (Kd) between BPA and ΔDNA-PKcs #6 using Scatchard analysis. As shown in Figure 2*C*, BPA bound to ΔDNA-PKcs #6 with a Kd of 137 nM. We next compared the protein yield of the DNA-PKcs WT and the DNA-PKcs mutants after affinity purification using BPA-immobilized beads. The protein yields of DNA-PKcs WT, #3, and #6 were estimated to be 1%, 3%, and 0.8% of the input, respectively. The BPA-binding activity of DNA-PKcs WT may be very similar to that of DNA-PKcs#6.

### Inhibition of DNA-PK kinase activity by BPA

**Figure 3:**
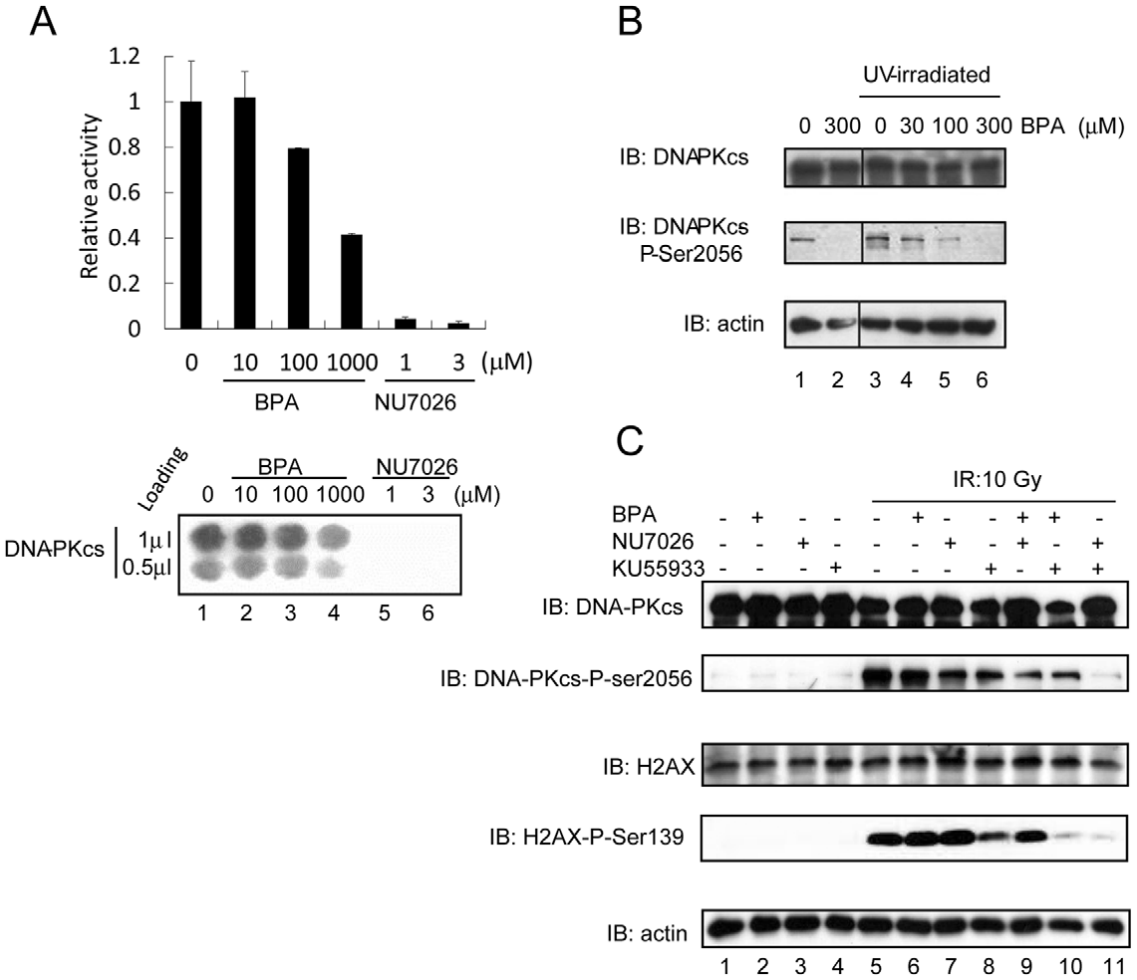
Inhibitory effect of DNA-PKcs kinase activity by BPA. (A) Protein kinase activity was assayed *in vitro* using the SignaTECT®. Purified DNA-PKcs, biotinylated p53-derived peptide substrate, DNA, and [γ-^32^P] ATP were mixed and BPA or NU7026 was added as indicated. Samples were incubated at 30°C for 5 min. Incorporation of ^32^P into the substrate was measured using an Imaging plate. (B) 293T cells were exposed to 150 J/m^2^ of 254 nm UV followed by incubation at 37°C for 6 h. Cells were harvested and immunoblot analysis was performed using antibodies to DNA-PKcs-phospho-serine 2056, total DNA-PKcs, and actin (loading control). BPA was added 1 h prior to UV-irradiation at the concentrations indicated. (C) M059K cells were exposed to 10 Gy of γ-rays. Cells were pre-incubated with BPA (300 μM, 3 h), Nu7026 (10 μM, 24 h) or Ku55933 (10 μM, 1 h) separately or in combination. One hour after irradiation, cells were harvested and the amount of DNA-PKcs, DNA-PKcs-phospho-serine 2056, H2AX, H2AX-phospho-serine 139, and actin (loading control) were analyzed by immunoblotting.

To examine possible effects of BPA on DNA-PK function, we first measured DNA-PK kinase activity using an *in vitro* DNA-PK assay system. Treatment with 100 μM and 1000 μM BPA decreased DNA-PK kinase activity by 21% and 59%, respectively, compared with that observed for the untreated form (Fig. 3*A*). NU7026, an inhibitor of DNA-PKcs [20], was used as a positive control. DNA-PKcs binds to (and requires) the ends of DNA DSBs to phosphorylate a number of nuclear proteins, including itself [14]. To examine the effects of BPA on DNA-PK kinase activity in cultured cells, autophosphorylation of DNA-PKcs at Ser 2056 after UV irradiation [21] was analyzed in 293T cells. DNA-PKcs autophosphorylation at Ser 2056 decreased in a manner that was dependent on BPA concentration (Fig. 3*B*). Next, M059 K cells were treated with IR instead of UV, and the phosphorylation of DNA-PKcs at Ser 2056 and H2AX at Ser 139 was examined. H2AX-ser139 (γ-H2AX) is mainly phosphorylated by Ataxia Telangiectasia Mutated protein (ATM) in response to DSBs [22], and any residual phosphorylation in the absence of ATM is due to DNA-PK [22]. BPA did not affect the phosphorylation of Chk2 (a downstream substrate of ATM) or Chk1 (a substrate of another related kinase, ATR (ATM- and Rad3-related) in response to DNA damage (Fig. *S1*). Therefore, we next examined the effects BPA in combination with KU55933, which is an inhibitor of ATM [23], on IR-induced phosphorylation at these sites. The phosphorylation of DNA-PKcs at Ser 2056 was partially, but markedly reduced by NU7026 or KU55933 and was abolished by combined treatment with NU7026 and KU55933. This result indicated that IR-induced phosphorylation of DNA-PKcs at Ser2056 in M059K cells might be mediated by both ATM and DNA-PKcs itself. BPA alone did not show any discernible effect on the phosphorylation status of DNA-PKcs at Ser 2056 (Fig. 3*C* lane 6), but it did decrease the level of residual phosphorylation in the presence of NU7026 or KU55933 (Fig. 3*C* lanes 9 and 10 compared to lanes 7 and 8 respectively). Figures 3*B and 3*C show that phosphorylation of DNA-PKcs was detected without IR treatment (although the signal in Fig. 3*C* is very low). Autophosphorylation of DNA-PKcs without treatment (Fig. 3*B* lane 1) was stronger than the autophosphorylation shown in Figure 3*C*. However, the basal level of DNA-PKcs autophosphorylation is likely to be dependent on cell-type (Fig. 3*B*; 293T cells, Fig. 3*C*; M059K cells). Phosphorylation of H2AX at Ser 139 was reduced by KU55933 (Fig. 3*C* lane 8). NU7026 did not show any discernible effect on the phosphorylation of H2AX at Ser 139 alone, but abolished any residual phosphorylation when combined with KU55933 (Fig. 3*C* lanes 7 and 11), which is in agreement with earlier studies [22]. BPA showed similar effects to those of NU 7026, i.e., BPA alone did not reduce H2AX Ser 139 phosphorylation, but showed synergistic effects when combined with KU 55933 (Fig. 3*C* lanes 6 and 10). BPA, like NU 7026, might inhibit DNA-PK activity in response to DNA damage in M059K.

### Molecular mechanism underlying the inhibition of DNA-PK function mediated by BPA

**Figure 4:**
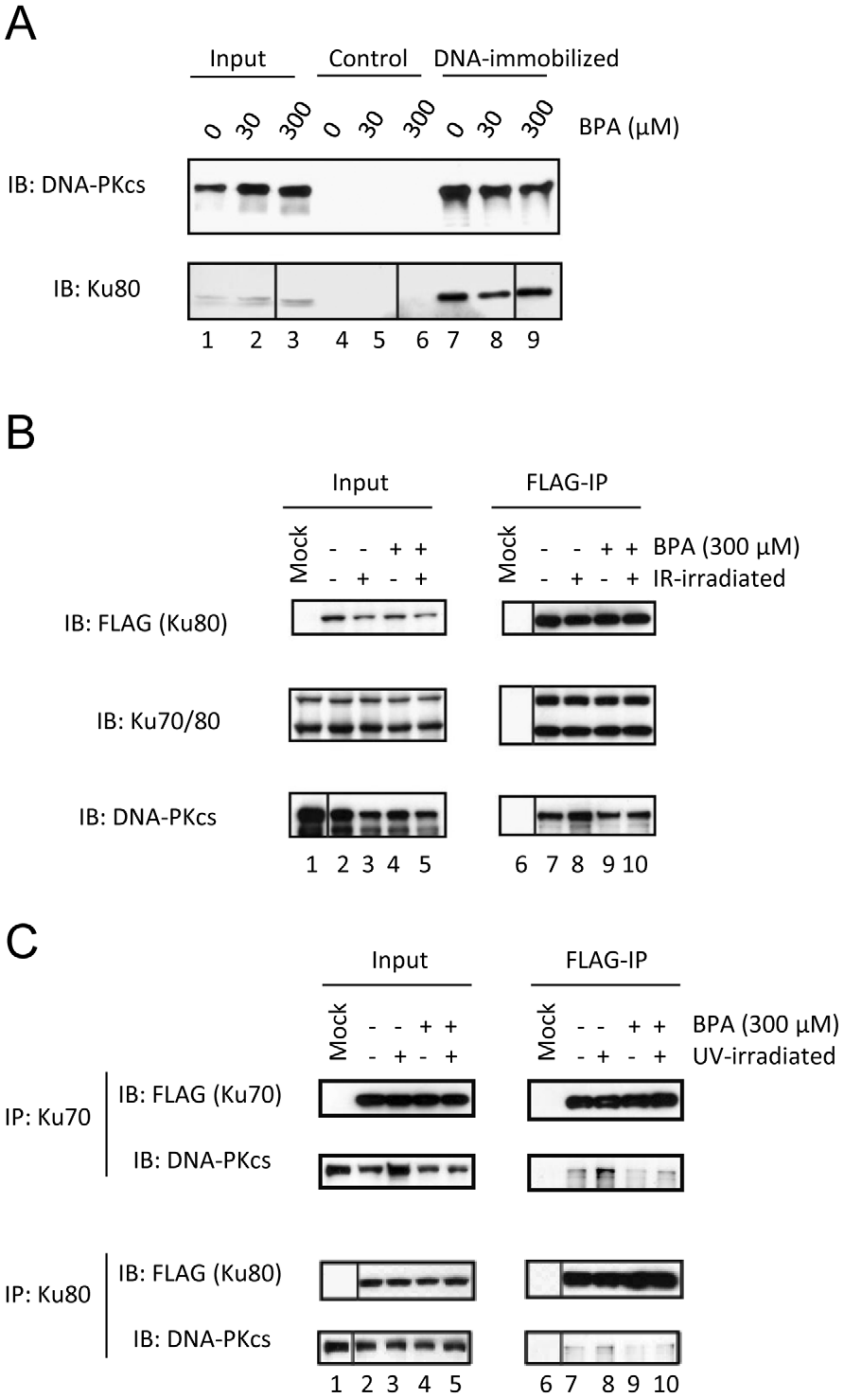
The inhibition of binding between DNA-PKcs and Ku-complexes by BPA. (A) M059K cells extracts were pre-incubated with the indicated concentrations of BPA and, thereafter, with DNA-immobilized beads or control beads. Bound proteins were eluted with SDS sample buffer and detected by immunoblotting. (B) (C) FLAG-Ku80 (B, bottom and C) or FLAG-Ku70 (B, top) was expressed in 293T cells incubated with 300 μM BPA and then irradiated with γ-rays (B) or UV (C). Cell lysates were immunoprecipitated using anti-FLAG agarose beads and immunoblotted with the indicated antibodies.

Next, to understand the mechanism underlying the inhibition of DNA-PK activity by BPA, we examined the effect of BPA on DNA-binding by DNA-PKcs. Double-strand DNA was covalently conjugated to FG beads and then incubated with extracts from M059K cells. In the absence of BPA, both DNA-PKcs and Ku80 were identified within the bound fraction of DNA-immobilized beads (Fig. 4*A* lane 7), although neither was found when control beads were used (Fig. 4*A* lanes 4–6). However, in the presence of BPA, binding of DNA-PKcs to the double-strand of DNA was decreased in a dose-dependent manner (Fig. 4*A* lanes 8 and 9). On the contrary, the interaction of Ku80 and double-strand of DNA was not affected by BPA up to a concentration of 300 μM (Fig. 4*A* lanes 8 and 9). Furthermore, we performed an electrophoretic mobility shift assay and confirmed that the signal of the DNA-PK (DNA-PKcs and Ku70/Ku80) and double strand DNA complex were reduced in the presence of BPA and the signal of Ku/DNA complex were slightly increased (Fig. *S2*). BPA is likely to affect the interaction between DNA-PKcs and Ku70/80 on DNA.

To examine the interaction between DNA-PKcs and Ku70/Ku80 more directly, we performed immunoprecipitation (IP) using FLAG-Ku70 or FLAG-Ku80 recombinants, which were expressed in 293T cells after IR-irradiation (Fig. 4*B*) or UV-irradiation (Fig. 4*C*). After IR-irradiation, co-precipitation of DNA-PKcs with Ku80 increased compared with that in the non-irradiated controls (Fig. 4*B* lanes 7 and 8). However, treatment with BPA decreased the amount of DNA-PKcs that co-precipitated with Ku80 (Fig. 4*B* lane 10). Similarly, UV-irradiation increased co-precipitation of DNA-PKcs (Fig. 4*C* lane 8), which was slightly decreased by BPA (Fig. 4*C* lane 10). These results suggested that BPA inhibits DNA-PK activity by interfering with the interaction between DNA-PKcs and Ku70/80.

### DNA-PKcs-dependent radiosensitization by BPA

**Figure 5:**
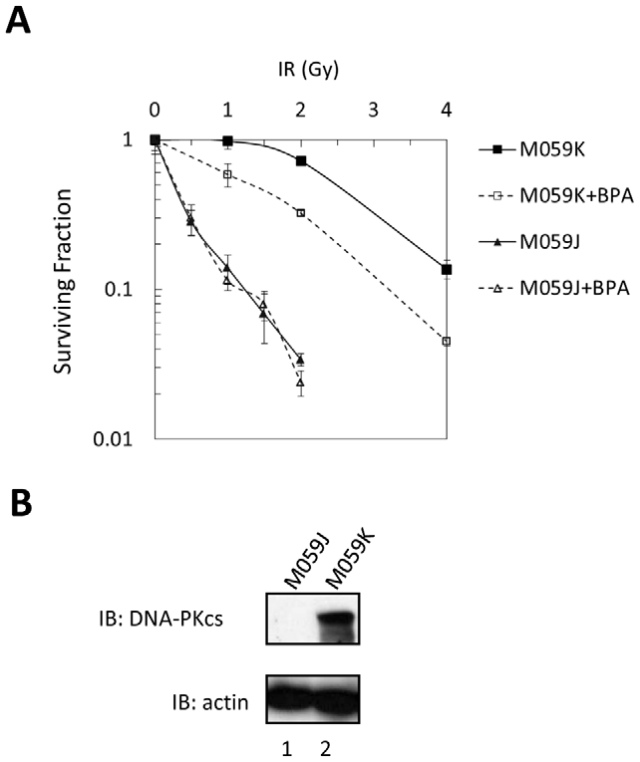
Effects of BPA in cultured cells. (A) Survival of logarithmically growing M059J and M059K cells after exposure to various doses of γ-rays as measured by colony formation. BPA was added to the culture media 3 h prior to irradiation and removed 2 h after irradiation. Cells on the survival curve “M059K+BPA” were treated with BPA at IR dose “0”. The results shown represent the mean from three independent experiments; bar, SE. (B) Expression of DNA-PKcs was detected in M059K, but not in M059J cells.

To further investigate the influence of BPA on DNA-PKcs function in cultured cells, we examined the effects of BPA on radiosensitivity in terms of cell killing in M059J (DNA-PKcs-deficient) and M059K (DNA-PKcs-proficient) cells (Fig. 5*B*). Cells were treated with 300 μM of BPA for 5 h (BPA was added 3 h prior to γ-irradiation and removed 2 h after irradiation). In agreement with a previous report by [16], M059J cells were more radiosensitive than M059K cells. Treatment with 300 μM of BPA increased the radiosensitivity of M059K cells, but not that of M059J cells (Fig. 5*A*). Thus, DNA-PKcs is a possible requirement for BPA-mediated effects on cell radiosensitivity.

## Discussion

BPA mediates a number of estrogenic effects, including enlargement of the prostate, by interacting with ER and other related steroid hormone receptors [1], [6]. BPA binds to other cellular proteins in addition to nuclear receptors [7]. The present study identified DNA-PKcs as a novel BPA-binding protein. BPA inhibited the kinase activity of DNA-PK in an *in vitro* kinase assay (Fig. 3*A*) and also suppressed phosphorylation of presumptive *in vivo* targets, i.e., DNA-PKcs itself, at Ser2056 and H2AX at Ser139 (Fig. 3*B, C*). We also demonstrated that BPA enhances the radiosensitivity of M059K cells, but not that of M059J cells (which are deficient for DNA-PKcs). The inhibitory effects on DNA-PK activity and radiosensitizing effects on cells were observed at high BPA concentrations, (≥100 μM). These results suggest that BPA affects DNA repair and/or recombination, especially at high doses.

The binding of DNA-PKcs to BPA was mediated by amino acids 927–1857, which form part of the HEAT repeats, and by amino acids 2755–3586, encompassing part of the FAT domain (Fig. 2). The latter region partly overlaps the Ku 70/80 heterodimer-binding domain (3002–3850) of DNA-PKcs, revealed by a previous study [20]. BPA inhibited the binding of DNA-PKcs to DNA, but did not affect the binding of Ku to DNA (Fig. 4 and Fig. S2). We also showed that BPA interfered with the IR-induced interaction between Ku and DNA-PKcs, however, this interference was less pronounced in the case of UV-irradiation. Arguably the UV-FLAG-IP data could be due to increased input. However, this increased input is not unexpected and may be a result of stabilized DNA-PKcs from the DNA-PK complexes formed after UV-induced DNA damage. BPA might inhibit the function of the DNA-PK complexes, at least in part, by interfering the interaction between DNA-PKcs and Ku70/80 on DNA although our study does not exclude the possibility that there are other inhibitory mechanisms of the DNA-PK complex by BPA.

Immunodeficient scid mice show defects in V(D)J recombination, increased radiosensitivity, and high susceptibility to lymphomagenesis. Although scid harbors a non-sense mutation in the Prkdc gene, resulting in the loss of only 2% of the carboxyl terminal region, the missing region encompasses the FATC domain, which is essential for kinase activity [20]. Moreover, disruption of the Prkdc gene results in a phenotype similar to that in scid mice. Thus, DNA-PKcs is indeed required for V(D)J recombination and repair of radiation-induced DSBs. However, although both V(D)J recombination and repair of radiation-induced DSBs require DNA-PKcs activity, a minimal level of activity is sufficient for V(D)J recombination [24]. As the inhibition of DNA-PK activity observed in the present study was ∼60% (even at the highest concentration of 1000 μM), the effects, if any, of BPA on V(D)J recombination are likely marginal.

Balb/c, 129 and C.B.17 mice harbor a variant Prkdc allele, resulting in two substitutions in the genomic DNA sequence and the translated protein sequence [25], [26]. DNA-PK activity and DNA-PKcs expression is not null in mice with this variant allele, but is reduced 5–10 fold compared with that in mice harboring the normal allele. The variant allele is associated with increased susceptibility to radiation-induced breast cancer and lymphoma. Additionally, variable levels of DNA-PK activity were reported in humans, and relatively low levels of DNA-PK activity are associated with an increased risk of lung, breast and cervical cancer [27], [28]. This indicates that a reduction in DNA-PK activity, even if there is residual activity, would result in an increased risk of cancer.

Several lines of evidence suggest the possible carcinogenic effects of BPA. *In vitro* studies show that BPA promotes the growth of neuroblastoma cells [29], and newborn rats exposed to BPA show increased susceptibility to prostate cancer when they become adults [30]. A study of mammary glands in the offspring of pregnant rats treated orally with BPA show that key proteins involved in signaling pathways, such as cellular proliferation, are regulated at the protein level by BPA [31].

It is also noted that a recent study using the nematode *Caenorhabditis elegans* demonstrated that BPA abrogates meiosis and increases embryonic lethality, which is associated with a failure of meiotic synapsis formation and chromosome instability [32]. The study further demonstrated germ-line specific down-regulation of genes involved in DSB repair, especially through homologous recombination, e.g., of mre-11, rad-54 and mrt-2. It is speculated that BPA affects the expression of these genes via steroid hormone receptors. Although the existence of similar regulatory mechanisms in mammals needs to be examined, this may be another mechanism through which BPA increases genetic instability leading to carcinogenesis.

Much attention has been focused on DNA-PK as a promising approach to developing new radiosensitizers. It was initially shown that wortmannin, a phosphatidylinositol 3-kinase inhibitor, sensitizes cultured cells to radiation by inhibiting DNA-PK [12]. The search for more specific inhibitors of DNA-PK led to the discovery of agents such as NU7026 and NU7441, which are highly selective for DNA-PKcs [20], [33]. Although the effects of BPA on other molecules remain to be clarified, the present study shows that BPA sensitizes cells to radiation in a DNA-PKcs-dependent manner.

Recently, the authorities in several countries have started to restrict the use of BPA because of its estrogenic effects, which can be observed at relatively low concentrations. The present results may also warrant the consideration of the possible carcinogenic effects of BPA, especially at high doses, mediated through its action on DNA-PK. We also observed that treatment of MCF7 cells with 300 μM of BPA for 24 h reduced cell viability to ∼20%, of that in untreated cells, and that this was accompanied by cleavage of PARP1, which is indicative of apoptosis (data not shown). We also found that BPA binds to GCN1, which associates with GCN20 and mediates activation of the eIF-2a kinase, GCN2, in amino acid-starved cells to inhibit translation initiation [34]. Furthermore, the cardiovascular, diabetic, schizophrenic, and neurotoxic effects of BPA were shown in experimental animals [35]. There may be no plausible explanation for these symptoms in terms of known BPA targets, Our data show that BPA has moderate affinity for DNA-PKcs (ΔDNA-PKcs #6; Kd  = 137 nM). According to a previous study, ERRγ binds strongly to BPA with a Kd of 5.5–5.7 nM [1]. Another target PDI binds BPA comparatively weakly, with a Kd of 22.6 μM [7]. Taken together, these data clearly show that DNA-PKcs is not an exclusive target of BPA. BPA may still target other molecules. Multiple target proteins, including DNA-PKcs, may compete for binding to BPA within cells. Furthermore, our previous study showed that the Kd for the interaction between thalidomide and its primary target, cereblon, is 8.5 nM, although the drug inhibits the function of cereblon in living cells at a concentration of 10–100 μM [36]. Therefore, it may be feasible that the Kd (137 nM) is not the same as the effective cellular dose (100–300 μM). A multifaceted approach is necessary to improve toxicity assessment for human health.

## Materials and Methods

### Reagents

BPA (CAS no. 80-05-7; purity grade >99%; Tokyo Kasei Kogyo Co. Ltd.; lot no. FGN01) was dissolved in ethanol at a concentration of 100 mM and stored at −20°C until use.

### Cell culture

293T, M059J, M059K, and MCF7 cells were purchased from the American Type Culture Collection (Rockville, MD). 293T, M059J, and M059K cells were maintained in Dulbecco's modified Eagle's medium (Invitrogen, Carlsbad, CA) supplemented with 10% fetal bovine serum (FBS). MCF7 cells were maintained in Roswell Park Memorial Institute 1640 medium (Invitrogen) supplemented with 10% FBS.

### Preparation of BPA-immobilized beads

The immobilization protocol is shown in Fig. 1*B*. Magnetic FG beads (5 mg), which were prepared as described previously [37], were incubated with 100 mM BPA and 1 M potassium carbonate in 1 ml of *N,N*-dimethylformamide (DMF) for 24 h at 60°C. After washing in DMF and water, the beads were stored in 50% methanol at 4°C.

### Affinity purification using BPA-immobilized beads

Nuclear extracts were prepared from MCF7 human breast cancer cells as described previously [19] and dialyzed against buffer D (20 mM HEPES (pH 7.9), 10% glycerol, 0.2 mM ethylenediaminetetraacetic acid [EDTA], 1 mM dithiotreitol [DTT], 0.5 mM PMSF, 0.1% NP-40, and 100 mM KCl). BPA-immobilized beads (0.2 mg) were incubated with MCF7 nuclear extracts (protein content, 1 mg) for 4 h at 4°C. After washing five times with 1 ml of buffer D, the bound proteins were eluted with SDS sample buffer. In some experiments, 100 μM or 300 μM BPA was added to the extracts before incubation with the beads. Protein bands were excised from the polyacrylamide gels, treated with trypsin, and analyzed using a mass spectrometer.

### Recombinant DNA-PKcs expression and binding assay

cDNAs encoding fragments of human DNA-PKcs (#1 to #7) were obtained by RT-PCR and inserted into the pCMV-tag2B vector (Novagen, Milwaukee, WI) containing a 5′ Flag-tag. Each recombinant DNA-PKcs protein was expressed in 293T cells and purified using anti-FLAG M2 agarose beads (Sigma, St Louis, MO) according to the manufacturer's instructions. For the binding assays, 0.2 mg of equilibrated BPA-immobilized beads were incubated with approximately 100 pmol of recombinant proteins for 4 h at 4°C. After washing three times with 500 μl of buffer D, the bound proteins were eluted with SDS sample buffer and analyzed by SDS-PAGE followed by immunoblotting with an anti-FLAG antibody (Sigma).

### Calculation of the dissociation constant (Kd)

BPA-immobilized beads (0.2 mg) were incubated with different concentrations of purified ΔDNA-PKcs #6 (25–400 nM) for 4 h at 4°C. The beads were then washed three times with buffer (20 mM HEPES-NaOH (pH 7.2), 1 mM MgCl2, 150 mM KCl, 15% v/v glycerol, and 1 mM DTT) and the bound proteins were eluted in SDS sample buffer and immunoblotted with an anti-FLAG antibody. The Kd was calculated by Scatchard plot analysis.

### *In vitro* DNA-PK kinase assay

Protein kinase activation was assayed using the SignaTECT® DNA-Dependent Protein Kinase Assay System (Promega, Madison, WI). Following the manufacturer's protocol, reactions (25 μl) containing purified DNA-PK [38], DNA-PK activation buffer, reaction buffer, a DNA-PK biotinylated p53-derived peptide substrate, 0.5 μCi [γ-^32^P] ATP (10 mCi/ml), and BPA or NU7026 at the concentrations indicated were incubated at 30°C for 5 min. Termination buffer was then added and 10 μl of each reaction mixture was spotted onto SAM^2^® capture membrane. After washing with 2 M NaCl, membranes were dried and ^32^P incorporation was measured using an Imaging plate.

### Analysis of DNA-damage-inducible phosphorylation

M059K human glioblastoma cells were grown to approximately 80–90% confluence and irradiated using a ^60^Co source at a dose rate of 1–2 Gy/min at room temperature to achieve a cumulative dose of 10 Gy for each experiment. Cells were harvested 1 h after irradiation. Alternatively, 293T cells were UV-irradiated (254 nm) at a dose of 10 J/m^2^/s to achieve a cumulative dose of 150 J/m^2^. Cells were harvested 6 h after irradiation. Whole cell lysates were prepared in RIPA buffer [39], suspended in SDS sample buffer, and analyzed by SDS-PAGE followed by immunoblotting with anti-DNA-PKcs (Thermo Scientific, Waltham, MA), anti-DNA-PKcs-phospho-ser 2056 (Abcam, Cambridge, MA), anti-H2AX (Cell Signaling Technology, Danvers MA), anti-H2AX-phospho-ser 139 (Cell Signaling Technology), anti-Chk1 (Cell Signaling Technology), anti-Chk1-phospho-ser 317 (Cell Signaling Technology), anti-Chk2 (Cell Signaling Technology), anti-Chk2-phospho-thr 68 (Cell Signaling Technology) and anti-β-actin antibodies (Santa Cruz Biotechnology, Santa Cruz, CA).

### Analysis of DNA-binding activity using DNA-immobilized beads

To evaluate the effects of BPA DNA-binding activity on DNA-PKcs and Ku70/80, M059K cells were pretreated with 300 μM of BPA for 2 h. Whole cell lysates were serially diluted with high-salt buffer (20 mM HEPES-NaOH (pH 7.9), 400 mM KCl, 1 mM EDTA, 1 mM EGTA, 10% v/v glycerol, 0.02% v/v Tween 20, 1 mM DTT, and 1 mM PMSF) and a fraction (15 μl) was mixed with 0.4 mg of DNA-immobilized beads suspended in 35 μl of binding buffer (20 mM HEPES-NaOH (pH 7.2), 1 mM MgCl2, 50 mM KCl, 15% v/v glycerol, and 1 mM DTT) as described previously [40]. After 4 h at 4°C, the beads were washed three times with binding buffer containing 150 mM KCl, instead of 50 mM, and the bound proteins were eluted with SDS sample buffer. The eluate was analyzed by immunoblotting with anti-Ku80 (Santa Cruz Biotechnology) and anti-DNA-PKcs antibodies (Thermo Scientific).

### Electrophoretic mobility shift assay (EMSA)

EMSA was performed by modification of a previously described procedure [41]. Purified DNA-PK (37 ng of Ku70, 31 ng of Ku80, and 87 ng of DNA-PKcs) (V581A, Promega Madison, WI) was incubated for 2 h on ice in the absence or presence of BPA or various antibodies (purified mouse IgG1 (Bethyl Laboratories, Inc., Montgomery, TX), anti-Ku70 antibody (Santa Cruz Biotechnology), and anti-DNA-PKcs antibody (Thermo Scientific)), in buffer containing 10 mM Tris-HCl (pH 7.5), 50 mM NaCl, 5% glycerol, 1 mM EDTA, 1 mM DTT, and 0.1 µg/µl bovine serum albumin. After incubation, Labeled double-stranded 32 bp DNA (0.2 ng) was added in the each reaction and incubated for 5 min at 25°C in the presence of 1 µg of supercoiled plasmid DNA (pBlueScript SK+) as a non-specific binding competitor. Then, the reactions were resolved by electrophoresis at 10 V/cm through a 4% polyacrylamide gel in 1×TGE buffer (50 mM Tris-HCl (pH 8.5), 0.38 M glycine, and 2 mM EDTA). The gel was dried on a Whatman 3 MM paper and subjected to autoradiography.

The signal intensity of the radioisotope was quantified using ImageQuant software. The relative intensities of the ‘BPA-treated’ and ‘BPA-untreated’ signals generated by DNA-PK/DNA and Ku/DNA were determined after subtracting the background signal.

### Immunoprecipitation

To examine the interactions between DNA-PKcs and Ku70/80, FLAG-Ku70 or FLAG-Ku80 was expressed in 293T cells. Ku70 and Ku80 cDNAs were inserted into the pCMV-tag2B vector (Novagen) containing a 5′ Flag-tag. Cells were incubated with (0 or 300 μM) BPA and irradiated with UV or γ-rays as described above. Cell lysates were incubated with anti-FLAG agarose beads and bound material was eluted with SDS sample buffer. The eluate was analyzed by immunoblotting with anti-FLAG (Sigma), anti-Ku70/80 [42] and anti-DNA-PKcs antibodies.

### Measurement of cellular radiosensitivity using clonogenic assays

Cells were trypsinized at 37°C after irradiation and seeded onto 60-mm tissue culture dishes at a density expected to yield 10–100 colonies/dish. BPA (300 μM) was added to the culture medium 3 h prior to irradiation and removed 2 h post-irradiation. After an incubation period of up to 2 weeks, cells were stained with Giemsa and colonies comprising >50 cells were counted.

## Supporting Information

Figure S1 **BPA did not affect phosphorylation of Chk1 and Chk2.** The experiment was process as in Fig. 3*C*. Chk1 and Chk2 were analyzed by immunoblotting.
(TIF)

Figure S2 **BPA interfered the interaction between DNA-PKcs and Ku on DNA.** (A) End-labeled 32 bp DNA (0.2 ng) was incubated with or without purified DNA-PK (Ku70, Ku80, and DNA-PKcs) that were pre-incubated in the absence or presence of the indicated concentration of BPA, 1 µg of purified mouse IgG, 450 ng of anti-Ku70 antibody, or 450 ng of anti-DNA-PKcs antibody for 2 h. DNA-protein complexes were subjected to electrophoresis in 4% PAGE in TGE followed by autoradiography. Asterisk (*) indicates a nonspecific signal that was not discernibly supershifted with any antibody. (B) The relative signal intensities of DNA-PK/DNA and Ku/DNA with or without BPA treatment were calculated by densitometric measurement of the bands shown in Fig. S2A (lanes 2–4), and were expressed as arbitrary units by setting the values for ‘BPA-untreated’ DNA-PK/DNA and ‘BPA-untreated’ Ku/DNA to 1, respectively.
(TIF)
