# Supplementary figures and images for: Identification of DNA-Dependent Protein Kinase Catalytic Subunit (DNA-PKcs) as a Novel Target of Bisphenol A
Source: PLoS One. 2012 Dec 5;7(12):e50481. doi: 10.1371/journal.pone.0050481 (PMC3515620; doi:10.1371/journal.pone.0050481)

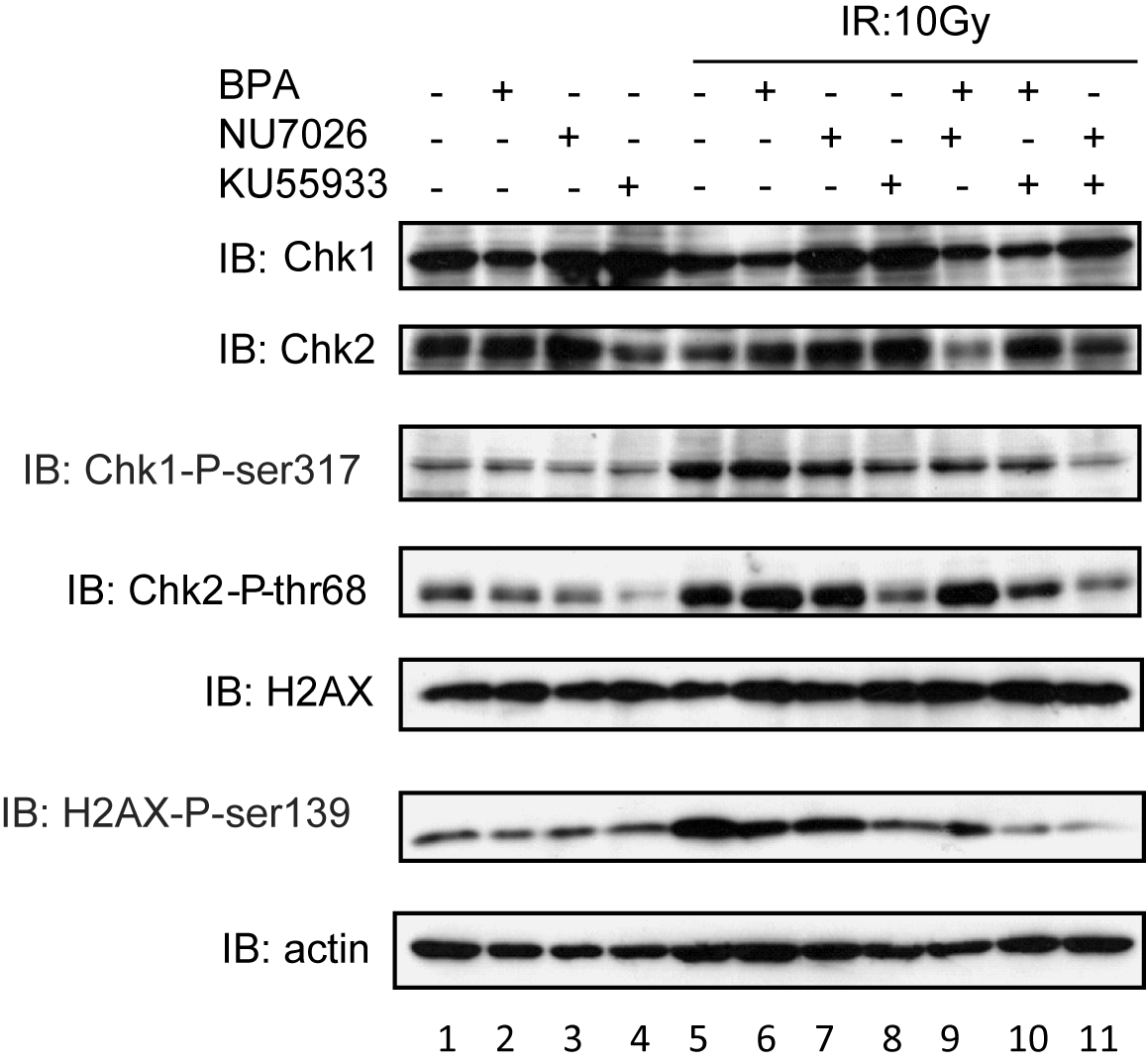

Supplement: Figure S1 — BPA did not affect phosphorylation of Chk1 and Chk2. The experiment was process as in Fig. 3C . Chk1 and Chk2 were analyzed by immunoblotting. (TIF) [file pone.0050481.s001.tif]

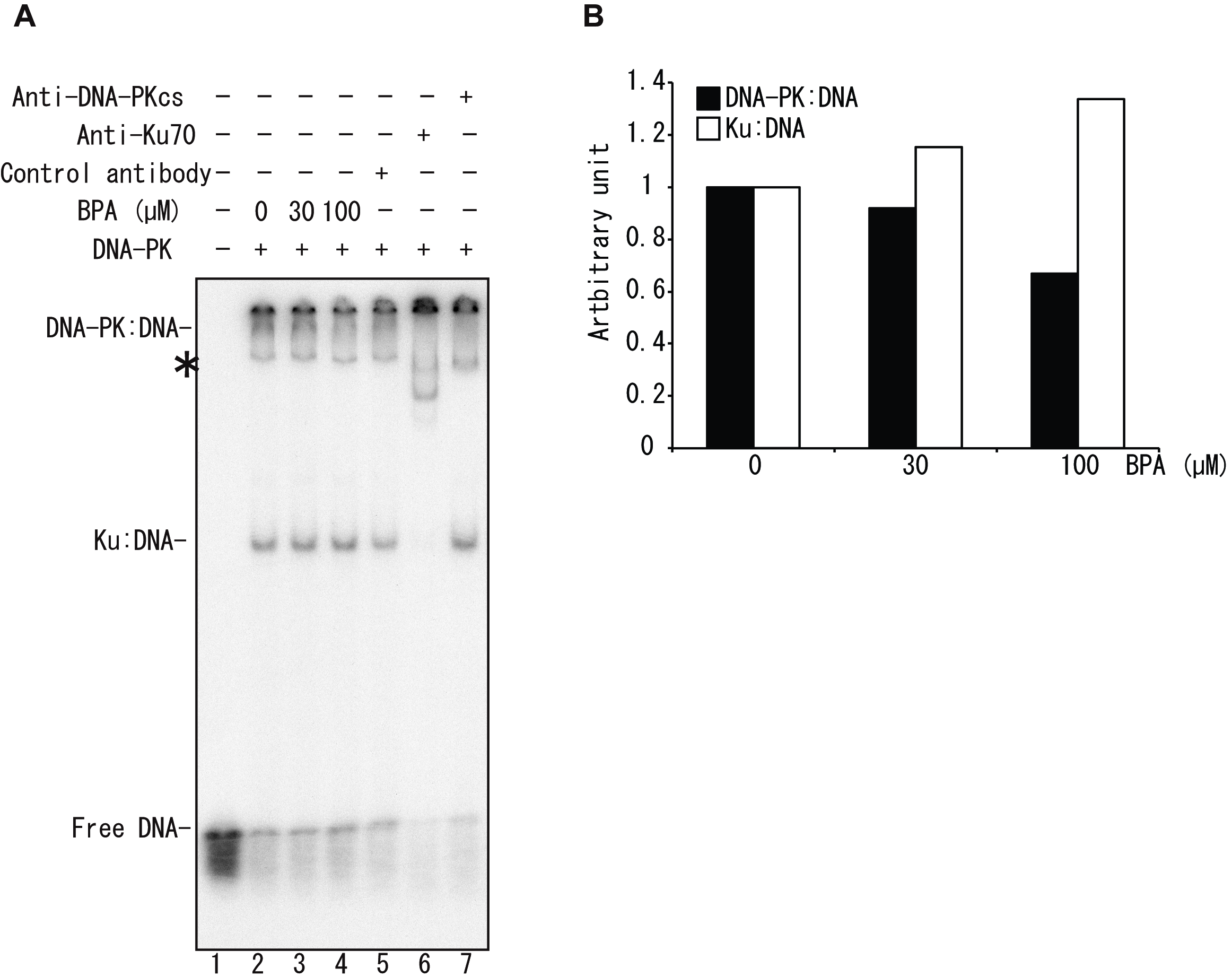

Supplement: Figure S2 — BPA interfered the interaction between DNA-PKcs and Ku on DNA. (A) End-labeled 32 bp DNA (0.2 ng) was incubated with or without purified DNA-PK (Ku70, Ku80, and DNA-PKcs) that were pre-incubated in the absence or presence of the indicated concentration of BPA, 1 µg of purified mouse IgG, 450 ng of anti-Ku70 antibody, or 450 ng of anti-DNA-PKcs antibody for 2 h. DNA-protein complexes were subjected to electrophoresis in 4% PAGE in TGE followed by autoradiography. Asterisk (*) indicates a nonspecific signal that was not discernibly supershifted with any antibody. (B) The relative signal intensities of DNA-PK/DNA and Ku/DNA with or without BPA treatment were calculated by densitometric measurement of the bands shown in Fig. S2A (lanes 2–4), and were expressed as arbitrary units by setting the values for ‘BPA-untreated’ DNA-PK/DNA and ‘BPA-untreated’ Ku/DNA to 1, respectively. (TIF) [file pone.0050481.s002.tif]
